# Ccn2a acts downstream of *cx43* to influence joint formation during zebrafish fin regeneration

**DOI:** 10.1242/bio.061674

**Published:** 2025-02-20

**Authors:** Victoria Hyland, M. Kathryn Iovine

**Affiliations:** Lehigh University, Department of Biological Sciences, Bethlehem, PA 18015, USA

**Keywords:** Connexin43, Cellular communication network factor 2 (Ccn2a), Β-catenin, Yap, Joint formation, Zebrafish, Skeletal regeneration

## Abstract

This study provides new insights into the molecular pathways dictating skeletal patterning during zebrafish fin regeneration. Connexin43 (Cx43) is known to influence skeletal patterning by inhibiting *evx1* expression and thereby regulating the timing of joint formation. Here, we demonstrate that *cellular communication network factor 2* (*ccn2a*) also contributes to this pathway. We find that Ccn2a appears to act downstream of Cx43 and similarly inhibits joint formation by inhibiting *evx1* expression. Pharmacological inhibition of β-catenin demonstrates that *ccn2a* is likely regulated by β-catenin. Additionally, this paper provides evidence that Yap signaling contributes to joint formation through regulating *ccn2a*. These findings provide novel insights into the role of Ccn2a during skeletal patterning.

## Teaser

**Summary:** This paper identifies Ccn2a, a multifunctional protein implicated in osteoarthritis, as a mediator of skeletal patterning during zebrafish fin regeneration.

## INTRODUCTION

Cellular communication network factor 2 (CCN2; also known as connective tissue growth factor, CTGF) is a multifunctional protein that can regulate several cell behaviors including proliferation, migration, adhesion, and differentiation (Tu et al., 2019; Nishida and Kubota 2020, Gonzalez and Brandan, 2019). CCN2 functions in the extracellular matrix (ECM) and modulates several signaling pathways, including TGFβ (Cheng et al., 2022) and Wnt signaling (Varghese et al., 2017). Further, CCN2 is downstream target of Yap signaling (Ott et al., 2003). Functionally, CCN2 has been implicated in osteoarthritis, a degenerative joint disease affecting joint structure and function (Tu et al., 2019; Yang et al., 2022). Unexpectedly, CCN2 has been implicated in both mediating and protecting against osteoarthritis (reviewed in Yang et al., 2022). Thus, further study into its function is warranted. Ccn2a, the zebrafish ortholog, has recently been discovered to have pivotal roles during regeneration of both the heart (Mukherjee et al., 2021) and larval spinal cord (Mokalled et al., 2016). First, *ccn2a* expression is induced following injury. In hearts, *ccn2a* regulates cardiomyocyte proliferation of the damaged tissue (Mukherjee et al., 2021), while in spinal cord *ccn2a* regulates the initial glial bridging process (Mokalled et al., 2016). These investigations suggest that Ccn2a may coordinate critical functions in tissue repair and developmental processes.

We use the zebrafish regenerating fin to provide insights into joint formation and skeletal patterning. Proper skeletal patterning, including the correct placement of joints, is essential for both form and function. However, the molecular pathways dictating skeletal patterning are not fully understood. Our research leverages this model to uncover the dynamics of joint formation, shedding light on regulatory mechanisms. The zebrafish caudal fin is comprised of 16-18 fin rays made up of bony segments flanked by joints. Following amputation, the fin regenerates rapidly. Regeneration begins with wound healing within the first 24 h post amputation (hpa), followed by the formation of a blastema containing the proliferative cells needed for outgrowth (reviewed in Wehner and Weidinger, 2015). The medially located blastema then becomes organized into a non-proliferative blastema located distally (i.e. the distal-most blastema, DMB) and the proliferative proximal blastema (Nechiporuk and Keating, 2002). Structurally, each fin ray is comprised of two hemirays of bony matrix which surrounds the medial mesenchyme. Cells responsible for building the bony segments and joints are also located laterally, and are called skeletal precursor cells (SPCs). Thus**,** SPCs reside in the lateral mesenchyme and differentiate into either osteoblasts or joint-forming cells (Tu and Johnson, 2011). The process of forming a new joint, or joint formation, begins when a band of joint-forming cells condense at the site of the future joint (Sims et al., 2009). We have shown that this ‘joint initiation’ step occurs at about 87 hpa (Dardis et al., 2017). Communication between the cells of the medial mesenchyme and the lateral SPCs allows for the proper timing of joint initiation, and therefore, appropriate patterning of the fin ray (Dardis et al., 2017; Bhattacharya et al., 2020). Outgrowth and differentiation proceed until regeneration is complete, about 3 weeks.

Connexin43 (Cx43) has been identified as an important player in skeletal patterning. Cx43 belongs to the connexin family of gap junction proteins, facilitating direct cell­–cell communication via the exchange of small molecules (<1500 Da) (Goodenough et al., 1996). Interestingly, hypomorphic mutations in *cx43* cause the *short fin* (*sof^b123^*) phenotype of short fins and short fin ray segments (Iovine et al., 2005). Further, gain of function mutations in the *cx43^lh10^* mutant cause longer fin ray segments (Bhattacharya et al., 2020). Prior findings indicate that Cx43 influences the observed differences in segment length by inhibiting joint-forming cell differentiation (Ton and Iovine, 2013). Thus, the short segment phenotype of *sof^b123^* is due to premature joint formation, and the long segment phenotype of *cx43^lh10^* is due to delayed joint formation.

The identification of molecular players acting downstream of Cx43 could provide insights into how the differentiation of joint-forming cells is regulated. For example, Cx43 was found to promote β-catenin signaling in the lateral SPCs, which in turn inhibits *evx1* expression (Bhattacharya et al., 2018). The *evx1* transcription factor is expressed specifically in joint-forming cells and is required for both the differentiation of joint-forming cells and for joint formation (Schulte et al., 2011; Ton and Iovine, 2013). Importantly, manipulation of Cx43 was sufficient to influence both the timing of *evx1* expression and the timing of joint formation (Dardis et al., 2017). These data strongly suggest that Cx43 regulates segment length by inhibiting *evx1* expression and, further, that periodic abrogation of Cx43 is required to permit *evx1* expression and joint formation. Identification of pathways acting upstream of Cx43 will elucidate how the alternating pattern of segment and joints is established (Seaver et al., 2023), while identification of pathways acting downstream of Cx43 will provide insights into how *evx1* expression and the timing of joint formation are regulated.

Motivated by the contribution of Ccn2a to regenerative processes such as the heart and spinal cord, and by its undefined role in osteoarthritis, we investigated its potential role in the regenerating fin. This paper explores the hypothesis that *ccn2a* functions in the Cx43-dependent joint formation pathway. Our results indicate that *ccn2a* acts downstream of *cx43* and suppresses *evx1* and joint formation. Furthermore, we found that *ccn2a* is downstream of β-catenin and Yap signaling. Therefore, Ccn2a acts downstream of Cx43 and β-catenin to suppress joint formation, and further implicates Yap signaling as a part of this pathway. These findings contribute to our understanding of the molecular pathway informing the timing of joint formation within the regenerating zebrafish fin.

## RESULTS

### *ccn2a* contributes to the Cx43-dependent joint formation pathway

**Fig. 1.**
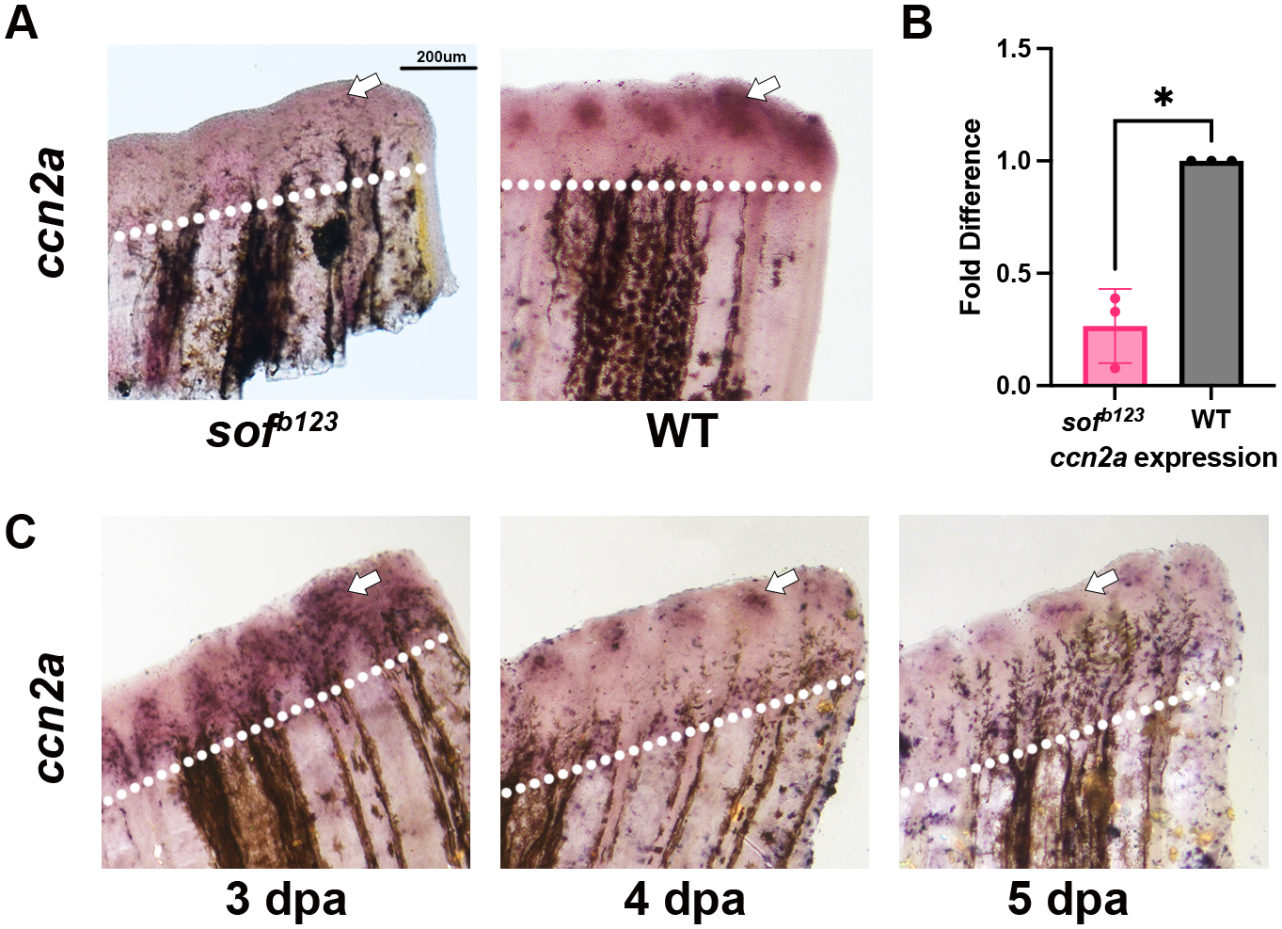
***ccn2a* is reduced in *sof^b123^* mutants and expression localizes to joints by 4 dpa.** (A) Fins were amputated at 50% and harvested at 5 dpa. Amputation planes are denoted by a white dotted line. *In situ* hybridization was performed using antisense digoxygenin-labeled probe against *ccn2a* to measure relative gene expression (*n*=4 per treatment group, with three biological replicates). (B) Reduction of gene expression was quantified through qPCR in both wildtype and *sof^b123^*mutants. Graph shows a mean±s.e.m. of three biological replicates fold difference (*n*=5 fins per replicate). A fold difference of 1 means no change from wild-type expression. Student's *t*-test (two tailed, unpaired) was used to assess significance with a *P*-value of 0.01. (C) Fins were amputated at 50% and harvested at 3 dpa, 4 dpa, and 5 dpa. Amputation planes are denoted by white dotted line. *In situ* hybridization was performed using antisense digoxygenin-labeled probe against *ccn2a* to measure relative gene expression (*n*=5 per treatment group, with three biological replicates). Scale bar: 200 µm.

Because *ccn2a* has been show to play an important role in zebrafish spinal cord regeneration (Mokalled et al., 2016) and heart regeneration (Mukherjee et al., 2021), we wondered if *ccn2a* may contribute to the process of joint formation during fin regeneration. Importantly, others have shown that expression of *ccn2a* is upregulated during fin regeneration and abrogated in post-regenerating fins (Mateus et al., 2015; Pfefferli and Jaźwińska, 2017). To test whether *ccn2a* may be part of the Cx43 pathway, we first monitored *ccn2a* mRNA levels in *sof^b123^* mutants. Indeed, *ccn2a* expression is reduced in *sof^b123^* regenerating fins compared to wildtype based on both *in situ* hybridization and quantitative reverse-transcriptase-PCR (qPCR) (Fig. 1A,B), suggesting that *ccn2a* may be downstream of *cx43*. Next, we followed *ccn2a* expression at different timepoints during fin regeneration. We observed that *ccn2a* expression appears to be broadly expressed at the distal ends of each fin ray at 72 h post amputation (hpa) but becomes more localized to the joint-forming cells by 4 days post amputation (dpa) (Fig. 1C). These results are consistent with other findings showing that *ccn2a* is expressed in a subset of lateral SPCs (Pfefferli and Jaźwińska, 2017). Since the *ccn2a* expression becomes restricted between 72 and 96 hpa, and joint initiation occurs at 87 hpa (Dardis et al., 2017), an interesting question is whether *ccn2a* contributes to the timing of joint formation.

**Fig. 2.**
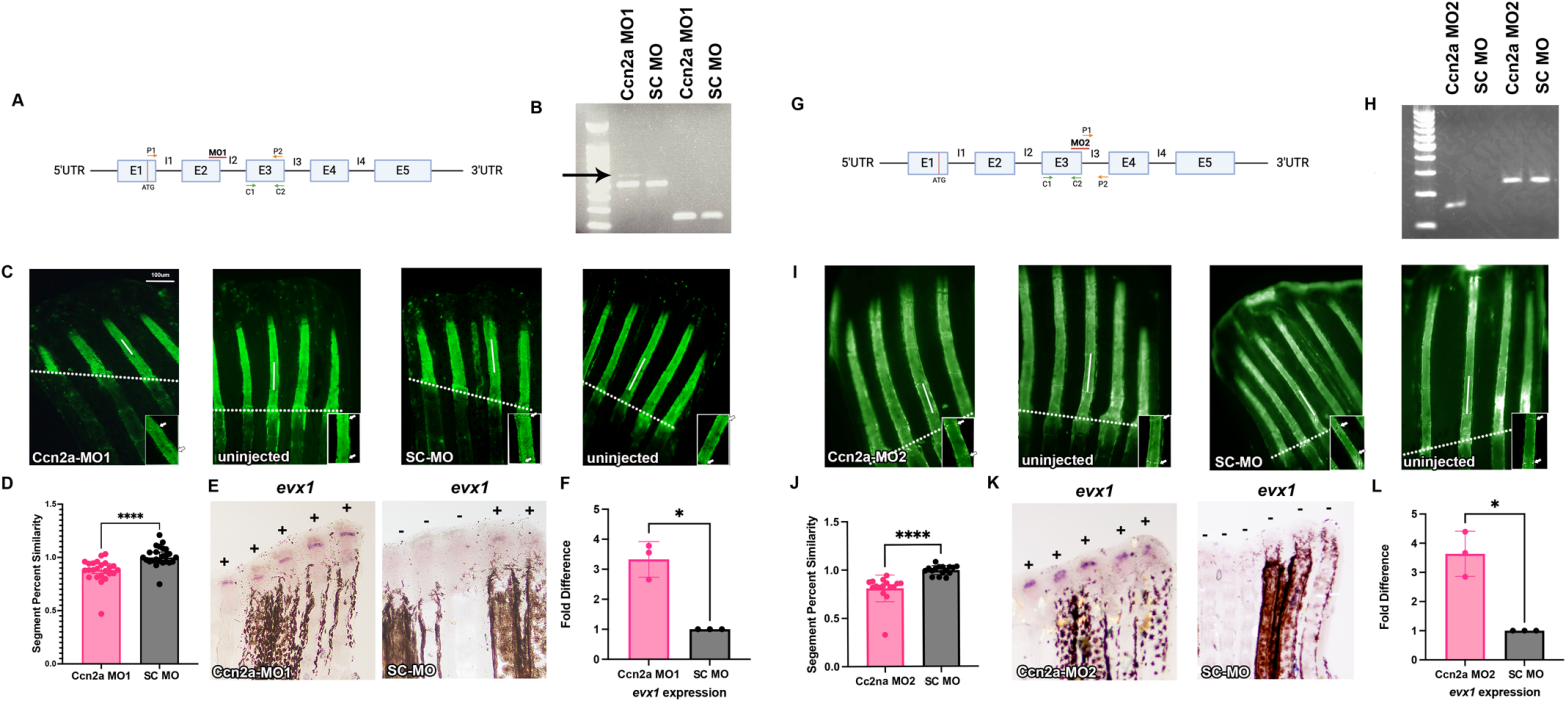
**Morpholino (MO)-mediated gene knockdown of Ccn2a reduces segment length in regenerating fins.** (A) Illustration of unspliced *ccn2a* mRNA with Ccn2a-MO1 binding site denoted by a red horizontal line. Primer locations shown used to determine whether *ccn2a* mRNA was targeted. (B) DNA gel showing amplicons using primer set P1 and P2. The slightly larger band (arrow) in Ccn2a-MO injected fins indicates that intron 2 was retained. The last two lanes show DNA amplified using control primers C1 and C2 (*n*=5 fins pooled together per cDNA, with three biological replicates). (C) Ccn2a-MO1 and SC-MO were injected into one side of the fin and compared to the uninjected side to calculate percent similarity. MO-injected fins were stained using calcein and measured for segment length (double white arrows). Representative images are shown and data are quantified using the percent similarity method (*n*=24 per treatment group, with two biological replicates). Insets identify individual segments, joints are indicated by white arrows. (D) Graph displays mean±s.e.m. of percent similarity and showed a significant decrease in segment length compared to SC-MO (two tailed, unpaired Student's *t-*test *P*<0.0001). (E) *In situ* hybridization was performed using an antisense digoxygenin-labeled probe against *evx1* to measure gene expression. Expression of *evx1* is measured by the frequency of positive or negative expression in fin rays denoted by a plus or minus sign. There are more *evx1* positive fin rays in Ccn2a-MO1 injected fins (*n*=5 per treatment, with three biological replicates). (F) Increase of gene expression was quantified through qPCR in both Ccn2a-MO1 and SC-MO. Graph shows a mean±s.e.m. of three biological replicates fold difference (*n*=5 fins per replicate). A fold difference of 1 means no change from wild-type expression. Student's *t*-test (two tailed, unpaired) was used to assess significance with a *P*-value of 0.02. (G) Cartoon illustration of unspliced *ccn2a* mRNA with Ccn2a-MO2 binding site denoted by a red horizontal line. Primer locations shown used to determine if *ccn2a* mRNA was targeted. (H) DNA gel showing amplicons using primer set P1 and P2 for MO2 (*n*=5 fins pooled together per cDNA, with three biological replicates). (I) Ccn2a-MO2 and SC-MO were injected into one side of the fin and compared to the uninjected side to calculate percent similarity. MO-injected fins were stained using calcein and measured for segment length (white arrows). Representative images are shown and data are quantified using the percent similarity method (*n*=24 for each treatment with two biological replicates). Insets identify individual segments, joints are indicated by white arrows. (J) Graph displays mean±s.e.m. of percent similarity and showed a significant decrease in segment length compared to SC-MO (two tailed, unpaired Student's *t-*test *P*=<0.0001). (K) *In situ* hybridization was performed using an antisense digoxygenin-labeled probe against *evx1* to measure gene expression. Expression of *evx1* is measured by the frequency of positive or negative expression in fin rays denoted by a plus or minus sign. There are more *evx1* positive fin rays in Ccn2a-MO2 injected fins (*n*=4 per treatment, with three biological replicates). (L) Increase of gene expression was quantified through qPCR in both Ccn2a-MO1 and SC-MO. Graph shows a mean±s.e.m. of three biological replicates fold difference (*n*=5 fins per replicate). A fold difference of 1 means no change from wild-type expression. Student's *t*-test (two tailed, unpaired) was used to assess significance with a *P*-value of 0.02. Scale bar: 100 µm.

**Fig. 3.**
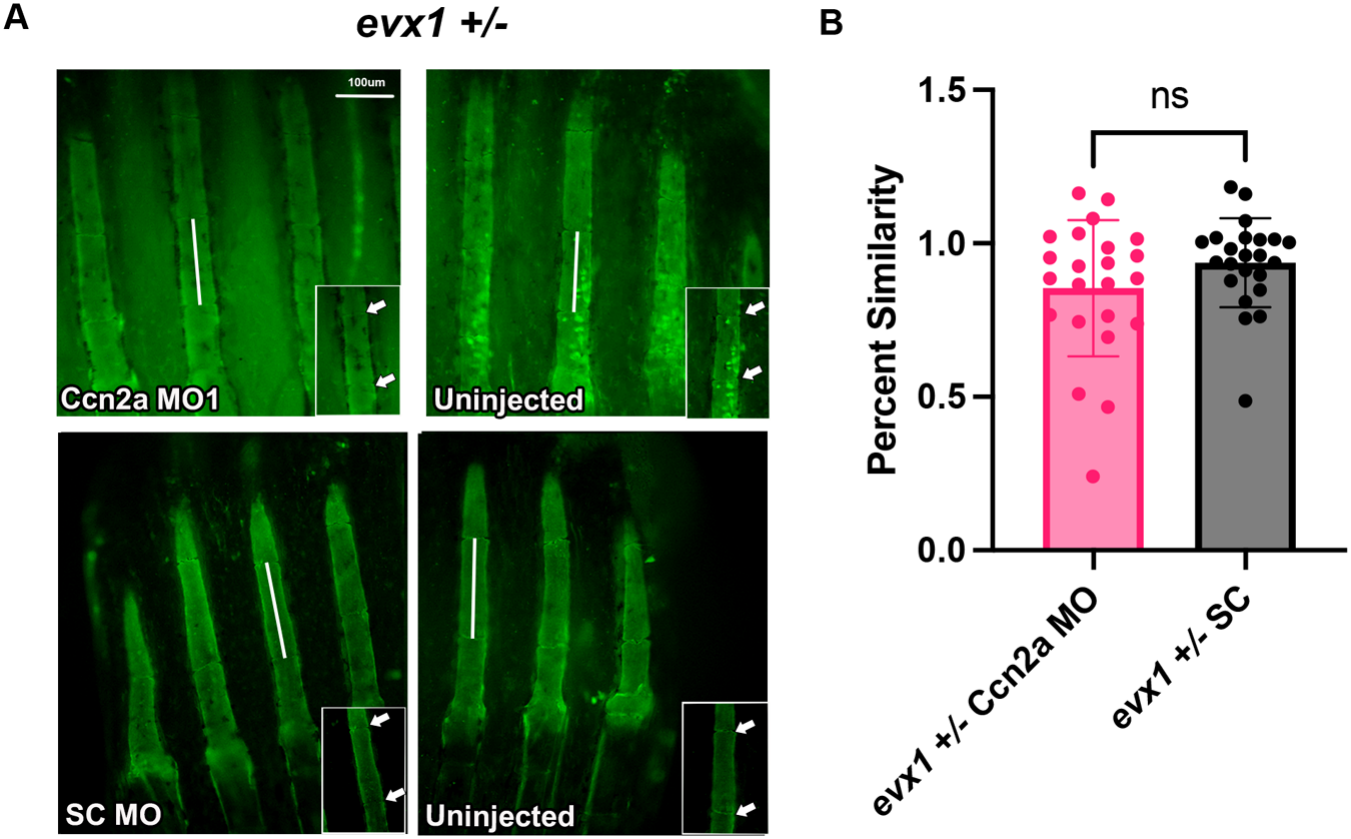
**Co-depletion of *evx1* and Ccn2a abrogates impacts of Ccn2a-KD on segment length.** (A) Ccn2a-MO1 or SC-MO were injected into the dorsal half of *evx1+/−* regenerating fins and compared to the uninjected side to calculate percent similarity. MO injected fins were stained using calcein and measured for segment length (white double arrows). Representative images are shown and data are quantified using the percent similarity method (*n*=27 for each treatment, with two biological replicates). Insets identify individual segments, joints are indicated by white arrows. (B) Graph displays mean±s.e.m. of percent similarity and showed there was no significant difference between Ccn2a-MO1 and SC-MO in *evx1+/−* fins (two tailed, unpaired Student's *t-*test *P*=0.15). Scale bar: 100 µm.

Next, to directly test if *ccn2a* influences the timing of joint formation (which we determine by measuring segment length), we completed morpholino (MO)-mediated gene knockdown (KD) (Thummel and Iovine, 2017) to inhibit Ccn2a function. We designed two splice-blocking MOs and confirmed that the MOs appropriately target *ccn2a* mRNA (Fig. 2A,G). Ccn2a-MO1 and Ccn2a-MO2 are predicted to block the removal of intron 2 and intron 3 respectively, which would result in nonfunctional proteins. Using reverse-transcription PCR (RT-PCR), we detected the retention of intron 2 in Ccn2a-MO1 treated fins, and we detected the retention of intron 3 in Ccn2a-MO2 treated fins. Neither intron was detected in the paired standard control MO (SC-MO) treated fins (Fig. 2B,H). We next tested whether Ccn2a-KD impacted the timing of joint formation by monitoring segment length (Fig. 2C,I). Fins were amputated at 50% and either MO was injected into half of the rays of the caudal fin at 72 hpa. Following injection, the fin was electroporated to induce cellular uptake of the MO. At 24 h post injection (hpi), fins were screened for fluorescein signal to detect the MOs, and segment length was measured at 7 dpa. We used the percent similarity method to compare treatments, which minimizes the impacts of fin-to-fin variation (as in Seaver et al., 2023). This method calculates the ratio of the injected side over the uninjected side, multiplied by 100 to get a percentage. Values that are close to 100% indicate little effect of the MO, while values far from 100% indicate the MO had an effect. We found that Ccn2a-KD has a percent similarity of about 88% for MO1 and about 80% for MO2, while the SC-MO treated fins have a percent similarity of about 100% (Fig. 2C,D,I,J). Thus, Ccn2a is necessary for the timing of joint formation. To verify that shortened segments are the result of elevated *evx1*, we next tested *evx1* levels in Ccn2a-MO treated fins via whole mount *in situ* hybridization. Fins were amputated at 50% and injected with either Ccn2a-MO or SC-MO at 72 hpa. Then, at 24 hpi, fins were harvested, fixed, and processed for *in situ* hybridization or qPCR. Indeed, we observed a significant increase in the percentage of *evx1-*positive fin rays treated for either Ccn2a-MO when compared to SC-MO treated fins (Fig. 2E,K). This result was verified as well through qPCR (Fig. 2F,L). Further, to confirm that Ccn2a and Evx1 functionally interact, we completed Ccn2a-KD in *evx1^+/−^* heterozygote mutants. These mutants have half as much Evx1 protein, but still produce fin ray segments (unlike *evx1^−/−^* homozygotes, Schulte et al., 2011). If reduced segment length in Ccn2a-KD fins is due to the subsequent increase in Evx1 function, this effect should be abrogated when Evx1 function is reduced. Indeed, segment length in *evx1^+/−^* fins treated for Ccn2a-KD is not significantly different from segment length in *evx1^+/−^* fins treated for SC-KD, revealing that the impact of Ccn2a-KD relies on appropriate levels of Evx1 function (Fig. 3). Together, these findings demonstrate that Ccn2a regulates the timing of joint formation by inhibiting *evx1*.

### *ccn2a* works downstream of β-catenin to cause skeletal phenotypes

**Fig. 4.**
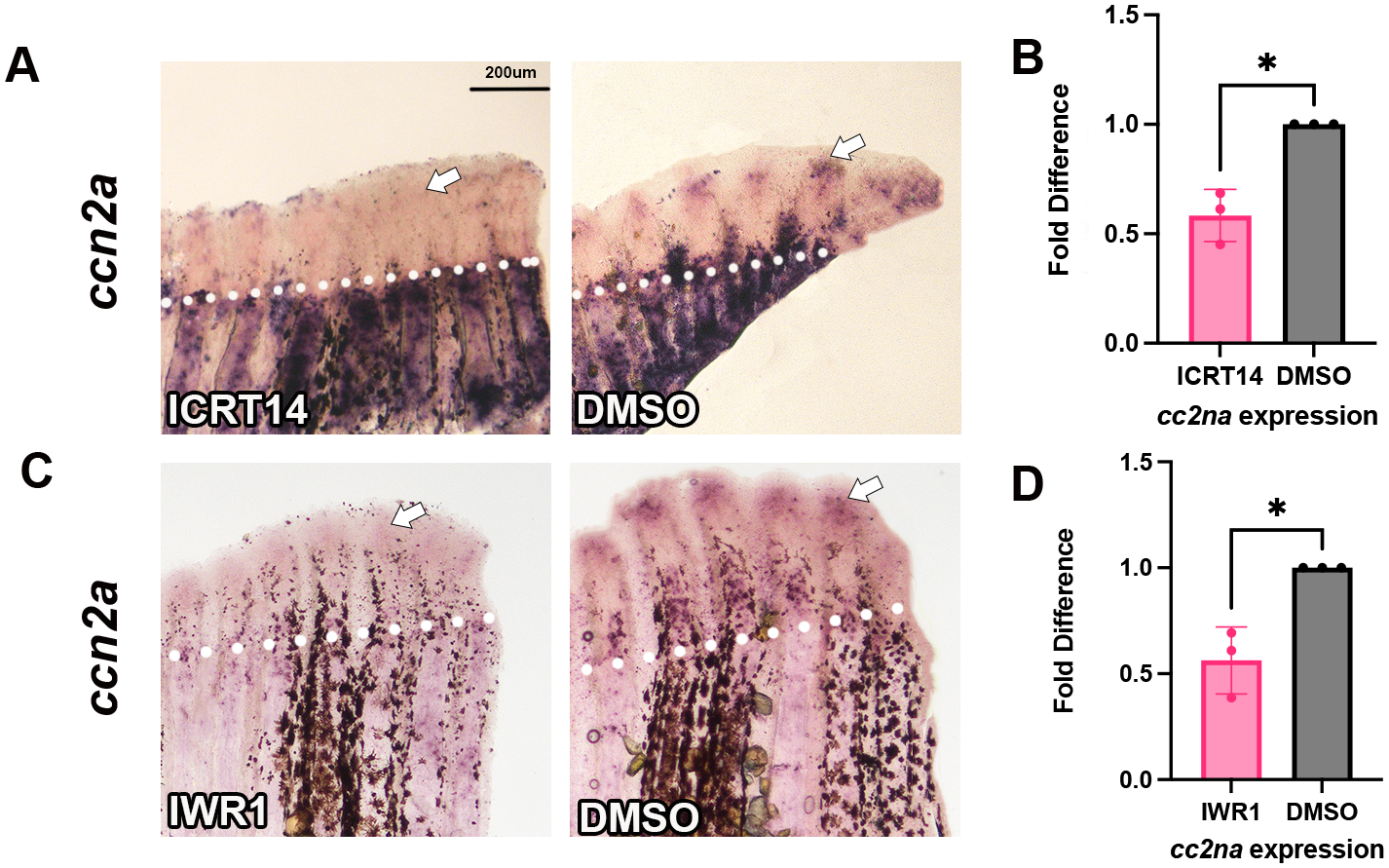
***ccn2a* expression was decreased with β-catenin inhibition.** Fins were amputated at 50% and treated with either 5 µM ICRT14 via injection or 10 µM IWR1 introduced into the water at 72 hpa. Fins were harvested 24 h later at 96 hpa. Amputation planes are denoted by white dotted line. *In situ* hybridization was performed using antisense digoxygenin-labeled probe against *ccn2a* to measure relative gene expression. (A) There was a decrease in *ccn2a* expression in ICRT14 treated fins compared to DMSO. (B) Reduction of gene expression was quantified through qPCR in ICRT14 treated fins. Graph shows a mean of three biological replicates fold difference and standard deviation (*n*=5 fins per replicate). A fold difference of 1 means no change from wild-type expression. The Student's *t*-test (two tailed, unpaired) was used to assess significance. (C) There was a decrease in *ccn2a* expression in IWR1 treated fins compared to DMSO. (D) Reduction of gene expression was quantified through qPCR in IWR1 treated fins. Graph shows a mean of three biological replicates fold difference and standard deviation (*n*=5 fins per replicate). A fold difference of 1 means no change from wild-type expression. The Student's *t*-test (two tailed, unpaired) was used to assess significance. Scale bar: 200 µm.

We found that β-catenin contributes to the timing of joint formation by acting downstream of Cx43, and in SPCs, to inhibit *evx1* (Bhattacharya et al., 2018). Interestingly, β-catenin has been shown to regulate *ccn2a* expression in pathways such as fibrogenesis (Varghese et al., 2017) and spinal cord degeneration (Gonzalez and Brandan, 2019). Therefore, we next tested if *ccn2a* may be downstream of β-catenin within the joint formation pathway. To test this possibility, fins were treated with either of two pharmacological inhibitors of β-catenin, IWR1 or ICRT14. IWR1 inhibits β-catenin through the stabilization of the destruction complex allowing for more efficient destruction of β-catenin (Chen et al., 2009). ICRT14 works through directly inhibiting β-catenin's interaction with TCF/LEF to prevent transcription of downstream targets (Gonsalves et al., 2011). Fins were treated at 72 hpa (pre-joint initiation) and evaluated at 96 hpa (24 h later, post-joint initiation) using both *in situ* hybridization and qPCR. Importantly, *ccn2a* expression was significantly decreased in response to both IWR1 and ICRT14 (Fig. 4). This supports the hypothesis that *ccn2a* expression is regulated downstream of β-catenin.

### Yap inhibition contributes to regulating the timing of joint formation

**Fig. 5.**
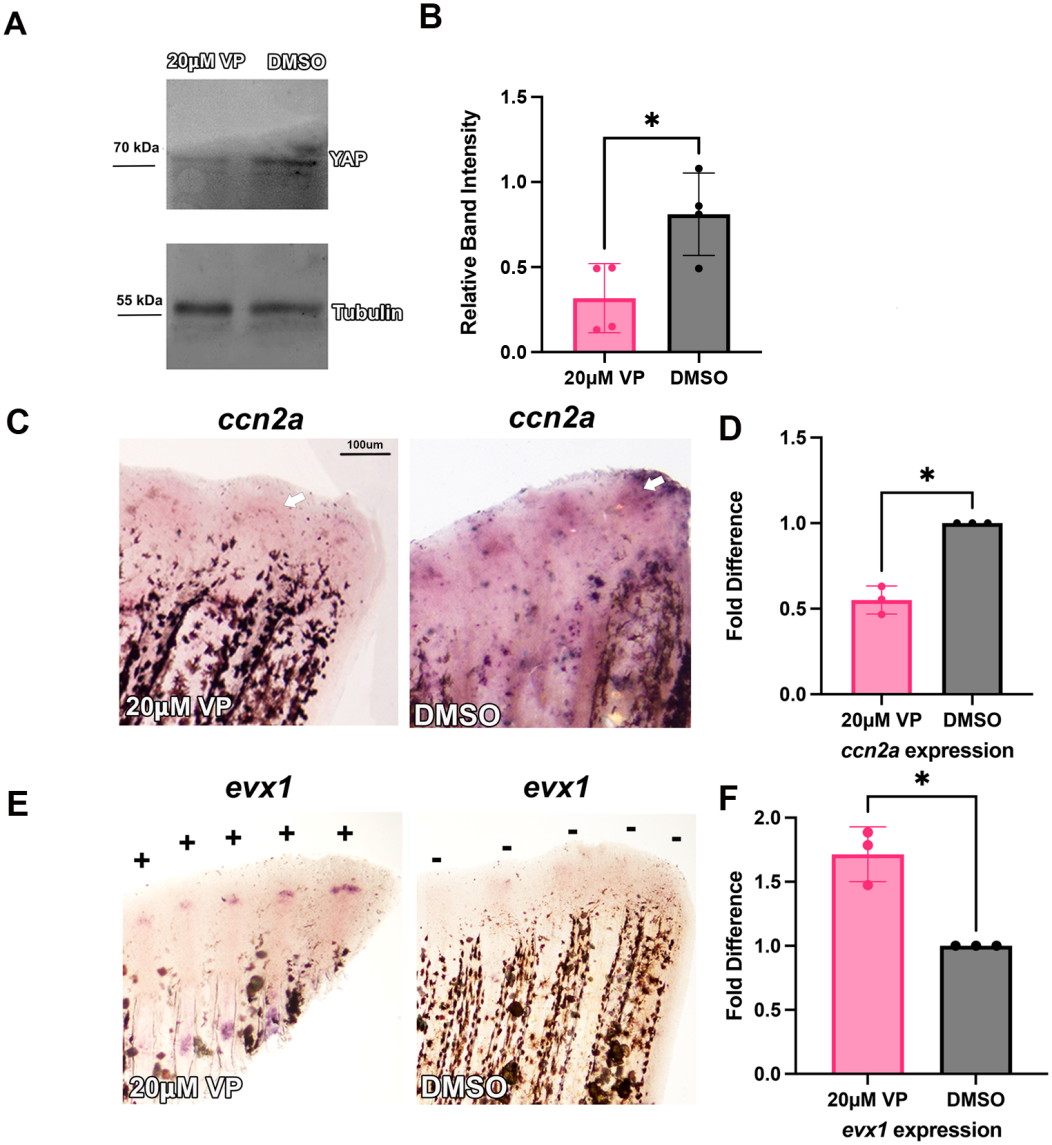
**Verteporfin inhibits Yap protein expression and alters *ccn2a* and *evx1* expression.** (A) Immunoblotting demonstrates reduced Yap protein expression in zebrafish fin lysates treated with 20 µM Verteporfin (VP) compared to DMSO-treated controls. Protein lysates were extracted from five pooled fins per treatment group. Tubulin was used as a loading control to normalize protein levels. (B) Quantitative analysis of Yap protein expression in zebrafish fins treated with 20 µM Verteporfin (VP) compared to DMSO-treated controls. Band intensity was measured using ImageJ and normalized to Tubulin as a loading control. Data represent the average Yap expression from four independent western blots, each performed using protein lysates from five pooled fins per treatment group. Statistical analysis (two tailed, unpaired Student's *t-*test *P* value=0.02) indicates a significant reduction in normalized Yap expression in VP-treated samples compared to controls. (C) Fins were amputated at 50% and treated with 20 µM Verteporfin at 72 hpa via injection. Fins were harvested 24 h later at 96 hpa. Amputation planes are denoted by white dotted line. *In situ* hybridization was performed using antisense digoxygenin-labeled probe against *ccn2a* to measure relative gene expression (*n*=5 for each treatment, with three biological replicates). There is less expression in treated fins when compared to DMSO shown with white arrows. (D) Reduction of *ccn2a* gene expression was quantified through qPCR in both DMSO and Verteporfin treated fins. Graph shows a mean of three biological replicates fold difference and standard deviation (*n*=5 fins per replicate). A fold difference of 1 means no change from wild-type expression. The Student's *t*-test (two tailed, unpaired) was used to assess significance with a *P*-value of 0.01. (E) *In situ* hybridization was performed using antisense digoxygenin-labeled probe against *evx1* to measure relative gene expression. There is an increase in positive fins rays for *evx1* with verteporfin treated fins (*n*=5 for each treatment with three biological replicates). Increase in *evx1* gene expression was quantified through qPCR in both DMSO and Verteporfin treated fins. (F) Graph shows a mean of three biological replicates fold difference and standard deviation (*n*=5 fins per replicate). A fold difference of 1 means no change from wild-type expression. The Student's *t*-test (two tailed, unpaired) was used to assess significance with a *P*-value of 0.02. Scale bar represents 100 µm.

**Fig. 6.**
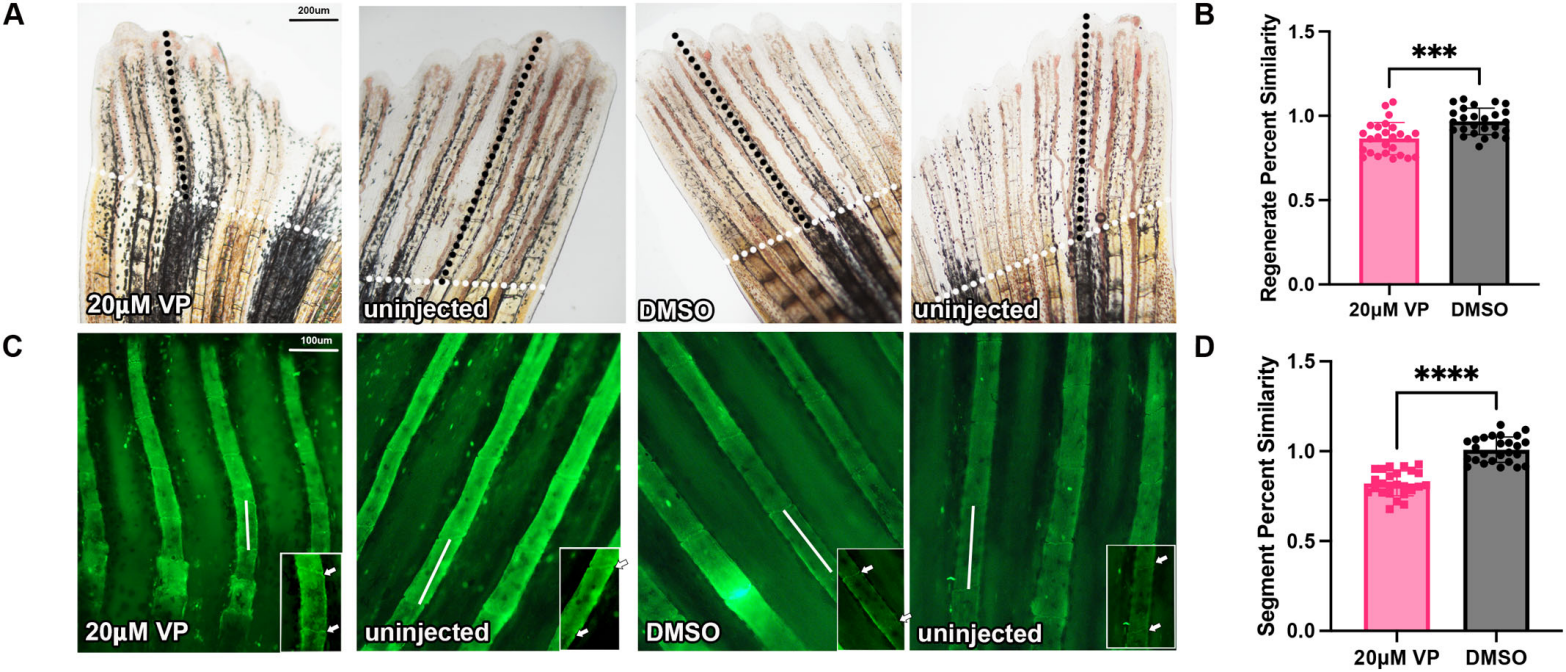
**Yap inhibition reduced segment length in regenerating fins.** (A) Verteporfin (VP) and DMSO injected fins were calcein stained and measured for regenerate length, indicated by black dotted lines (amputation plane is identified by white dotted lines). Verteporfin or DMSO was injected into one side of the fin and compared to their uninjected side to calculate percent similarity (*n*=26 for each treatment) with two biological replicates. (B) Graph displays mean±s.e.m. of percent similarity and showed a significant decrease in regenerate length compared to DMSO (two tailed, unpaired Student's *t-*test *P*<0.0001). (C) Verteporfin and DMSO injected fins were calcein stained and measured for segment length indicated by white lines and arrows. Verteporfin and DMSO were injected into one side of the fin and compared to their uninjected side to calculate percent similarity (*n*=26 for each treatment). Insets identify individual segments, joints are indicated by white arrows. (D) Percent similarity was calculated and showed a significant decrease in segment length compared to DMSO (two tailed, unpaired Student's *t*-test *P*=0.0001). Scale bars: 200 µm for top images; 100 µm for bottom images.

The *ccn2a* gene is also a known target of the Yap signaling pathway (Moya and Halder, 2019). Therefore, we next tested whether inhibition of Yap leads to segment length defects via inhibition of *ccn2a* expression. Verteporfin, a pharmacological inhibitor of Yap, works by increasing a 14-3-3 protein, which sequesters and inactivates Yap in the cytoplasm (Vigneswaran et al., 2021). To confirm that Verteporfin effectively inhibits Yap activity, Yap protein levels were monitored by immunoblotting. Protein extracts from Verteporfin treated fins showed a marked reduction in Yap levels compared to controls, consistent with its sequestration and degradation in the cytoplasm (Fig. 5A,B). Next, *ccn2a* expression was evaluated in Verteporfin-treated fins. Verteporfin was injected into half of the caudal fin rays at approximately 72 hpa leaving the other half as an internal control. At 24 hpi, fins were harvested and *ccn2a* expression levels were measured to confirm Yap inhibition. Due to *ccn2a* being a direct target of Yap, *ccn2a* is expected to be significantly decreased when compared to DMSO. Indeed, a significant decrease in *ccn2a* levels was observed (Fig. 5C,D). Moreover, as expected when *ccn2a* is decreased we found that *evx1* levels were elevated in Verteporfin-injected fin rays (Fig. 5E,F). We next tested whether Yap inhibition influences joint formation. Percent similarity of the injected versus the uninjected side was calculated to determine whether the treatment had an effect on length compared to the DMSO control. We observed a significant reduction in regenerate length as well as segment length (Fig. 6). These findings support the conclusion that Yap signaling contributes to joint patterning through the inhibition of *ccn2a*.

### Yap works downstream of Cx43 and β-catenin

**Fig. 7.**
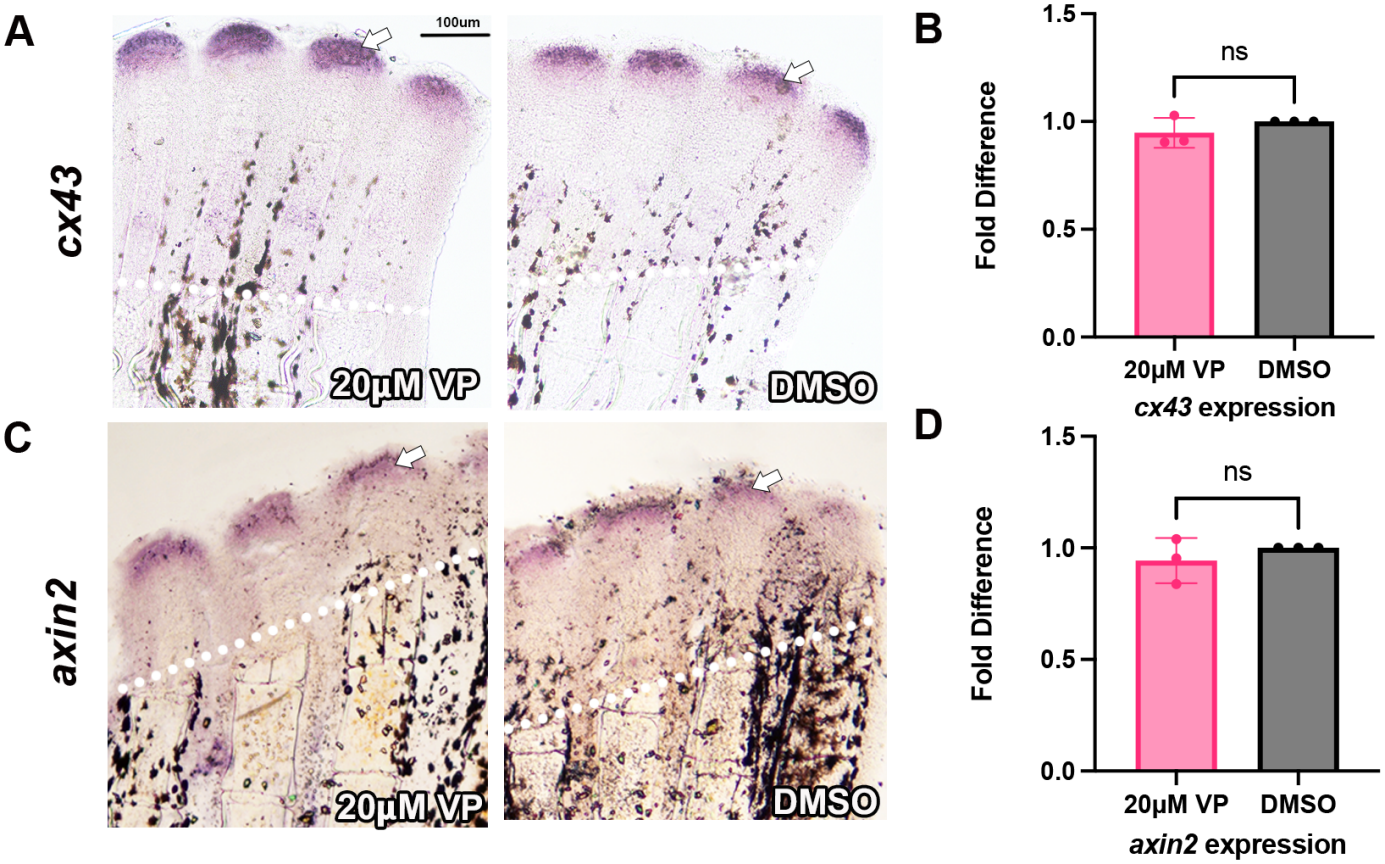
***cx43* and *axin2* expression are unchanged with Yap inhibition.** Fins were amputated at 50% and treated with 20 µM Verteporfin at 72 hpa via injection. Fins were harvested 24 h later at 96 hpa. Amputation planes are denoted by white dotted line. (A) *In situ* hybridization was performed using antisense digoxygenin-labeled probe against *cx43* to measure relative gene expression (*n*=4 for each treatment, with three biological replicates). (B) Gene expression was quantified through qPCR in both DMSO and Verteporfin treated fins. Graph shows a mean of three biological replicates fold difference and standard deviation (*n*=5 fins per replicate). A fold difference of 1 means no change from wild-type expression. The Student's *t*-test (two tailed, unpaired) was used to assess significance with a *P* value of 0.32. (C) *In situ* hybridization was performed using antisense digoxygenin-labeled probe against *axin2* to measure relative gene expression (*n*=4 for each treatment, with three biological replicates). (D) Gene expression was quantified through qPCR in both DMSO and Verteporfin treated fins. Graph shows a mean of three biological replicates fold difference and standard deviation (*n*=5 fins per replicate). A fold difference of 1 means no change from wild-type expression. The Student's *t*-test (two tailed, unpaired) was used to assess significance with a *P*-value of 0.43. Scale bar: 100 µm.

Since both Yap and β-catenin seem to impinge on *ccn2a* expression, the placement of Yap in the pathway is uncertain. To test whether Yap is upstream of *cx43*, *cx43* expression was assessed by *in situ* hybridization and qPCR in Verteporfin treated fins. We found no significant differences in *cx43* levels (Fig. 7A,B), indicating that Yap is not upstream of *cx43*. To test whether Yap is upstream of β-catenin, the expression of *axin2* was tested by *in situ* hybridization and qPCR, since *axin2* is a direct target of β-catenin signaling (Jho et al., 2002). There was also no significant changes in *axin2* levels when Yap was inhibited (Fig. 7C,D), indicating that Yap is not upstream of β-catenin. Together, these data suggest that Yap functions downstream or in parallel of β-catenin, and do not distinguish the possibilities that Yap functions as part of a common pathway with Cx43, or if Yap is activated independently. Future experiments will elucidate the contribution of Yap to the joint formation pathway.

## DISCUSSION

**Fig. 8.**
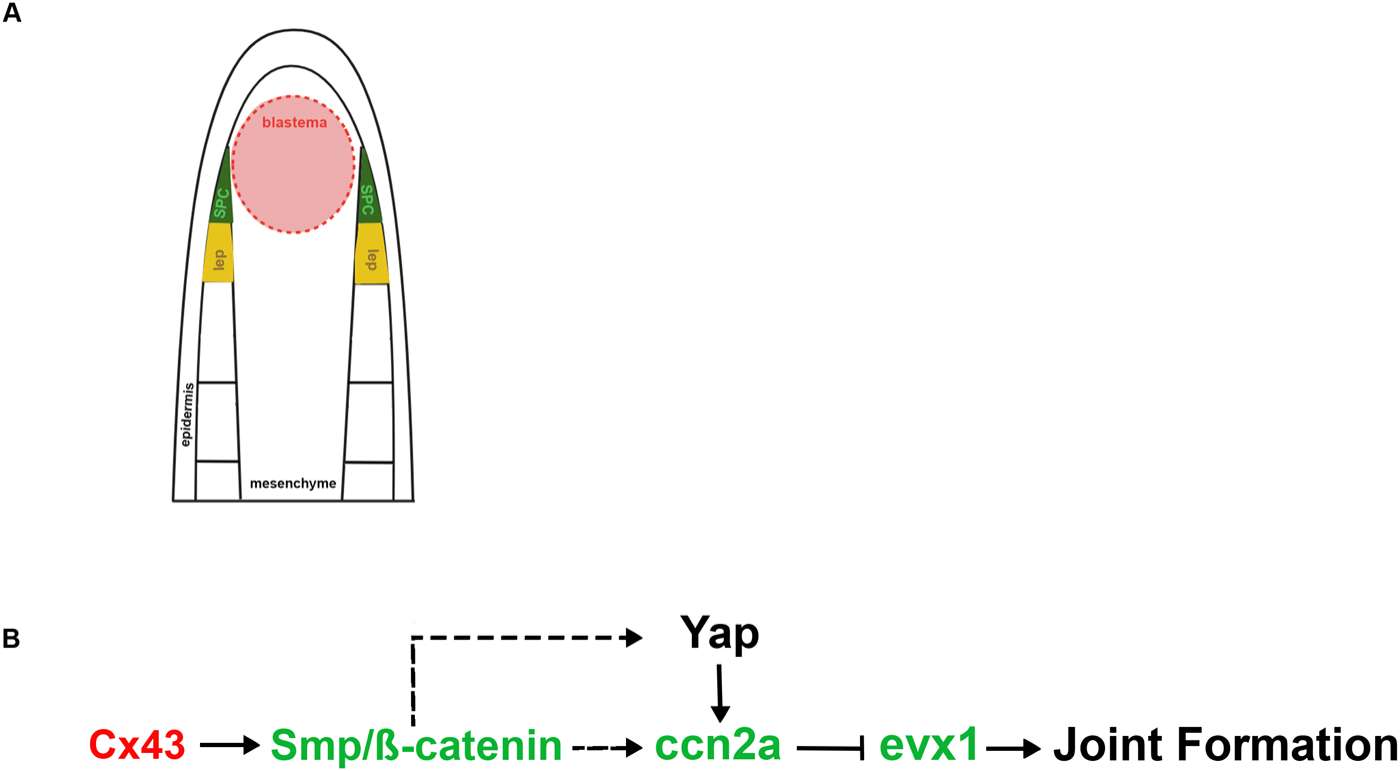
**Proposed model for interactions of *ccn2a* within the Cx43-dependent joint formation pathway.** (A) Schematic representation of a longitudinal section of a zebrafish fin ray. The blastema (red), located distally and medially, is composed of proliferative cells driving regeneration. Skeletal precursor cells (SPCs, green), situated laterally, will differentiate into either osteoblasts or joint-forming cells. Newly formed lepidotrichia, or the skeletal elements, are shown in yellow. (B) Cx43 (acting in the cells of the medial blastema, denoted by red text) promotes Smp/β-catenin (acting in the SPCs, denoted by green text), which may promote *ccn2a* expression (also in SPCs)*.* We show that Ccn2a inhibits *evx1*, which is required for joint formation. Yap may be regulated downstream of β-catenin or independently.

The findings presented in this study shed light on the molecular mechanisms governing the timing of joint formation during zebrafish fin regeneration, which depends on Cx43 activity. First, reduced *ccn2a* expression in *sof^b123^* mutants suggests that *ccn2a* expression is activated downstream of Cx43. Indeed, Ccn2a-KD caused reduced segment length and elevated *evx1* expression, demonstrating that Ccn2a contributes to the inhibition of joint formation. The expression of *ccn2a* in joint forming cells suggests that Ccn2a acts autonomously to influence *evx1* expression. Our investigation into the regulatory relationship between *ccn2a* and β-catenin provides additional insights into the molecular pathway governing joint formation. Pharmacological inhibition of β-catenin resulted in decreased *ccn2a* expression, suggesting that β-catenin may regulate *ccn2a* expression, which in turn modulates *evx1* levels. Based on these findings, we propose a model in which Cx43 activity promotes β-catenin signaling (shown in Bhattacharya et al., 2018), leading to the upregulation of *ccn2a* within skeletal precursor cells. Subsequently, *ccn2a* inhibits *evx1* expression, thereby inhibiting joint formation during zebrafish fin regeneration (Fig. 8). Periodic abrogation of Cx43 activity relieves the inhibition of *evx1* expression, permitting joint formation to occur (see also Dardis et al., 2017; Bhattacharya et al., 2020). These findings are significant because they demonstrate that the regenerating fin skeleton represents a suitable system to reveal mechanistic insights into Ccn2a function. Future studies may further enhance our understanding of the roles of Ccn2a in human joint disease, such as osteoarthritis.

The Hippo pathway has been found to contribute to fin regeneration. For example, manipulation of Yap signaling targets *ccn2a* (Mateus et al., 2015). Yap activity is regulated by changes in tension and cell density and was found to be active in the proliferative cells of the blastema of regenerating fins from 24-72 hpa (Mateus et al., 2015). We note this is at a time when *ccn2a* is more broadly expressed. By 4 dpa, *ccn2a* expression is restricted to the lateral mesenchymal compartment (Fig. 1). Moreover, Verteporfin treatment (which inhibits Yap) influences *ccn2a* expression, *evx1* expression, and segment length (Fig. 5). Thus, the Hippo–Yap pathway may also contribute to regulating the timing of joint formation. Furthermore, Yap has been shown to be regulated by β-catenin (Azzolin et al., 2014) and can interact with the β-catenin destruction complex. Therefore, β-catenin could potentially modulate Yap by releasing Yap from the destruction complex upon β-catenin activation (Azzolin et al., 2014). This model suggests that Yap, once activated by β-catenin, might subsequently influence the activity of *ccn2a*. This cascade of interactions hints at a complex network where Wnt signaling, β-catenin Yap, and Ccn2a collaborate to finely tune cellular the timing of joint formation.

At present, the precise molecular mechanisms underlying the interactions between Cx43, β-catenin, Yap, and *ccn2a* remain to be fully elucidated. Future studies will focus on untangling interactions between signaling pathways involved in regulating the timing of joint formation. Overall, our findings enhance our understanding of the molecular mechanisms governing joint formation and skeletal patterning, with potential implications for regenerative medicine and therapeutic interventions in skeletal disorders.

## MATERIALS AND METHODS

### Fish maintenance

Zebrafish (Danio rerio) males and females were maintained in circulating water system and kept at 27-28°C in a 14:10 h light:dark period (Westerfield, 2007). The quality of the fish tank water was monitored and dosed to maintain conductivity (400-600 mS) and pH (6.95-7.30). Research was performed according to the IACUC for Lehigh University (protocol #187, approved 3 July 2017). Food was provided to the zebrafish tanks twice daily. Every day, brine shrimp (hatched from INVE artemia cysts) was fed once and flake food once (Aquatox AX5) supplemented with 7.5% micropellets (Hikari), 7.5% Golden Pearl (300-500 μm, Brine Shrimp Direct) and 5% Cyclo-Peeze (Argent) (Banerji et al., 2017). Zebrafish used were between 6-15 months of age.

### Zebrafish strains

Wildtype, *sof ^b123^*, and *evx1/+* animals were used (Iovine and Johnson, 2000; Schulte et al., 2011). For amputation, fish were anesthetized in 0.1% tricaine solution and their caudal fin rays amputated to the 50% level. For *in situ* hybridization, regenerating fins were harvested and fixed in 4% paraformaldehyde (PFA) in phosphate-buffered saline (PBS) overnight at 4°C. After fixation, fins were dehydrated in 100% methanol and stored at −20°C.

### *In situ* hybridization

PCR products were used as templates for RNA transcription reactions; reverse primers included the binding site for T7 or T3 RNA polymerase (Table 1) Antisense digoxigenin-labeled probes were synthesized using T7 RNA polymerase for *ccn2a*, *cx43* and *axin2*, or T3 RNA polymerase for *evx1* (Roche). Whole-mount *in situ* hybridization was performed on harvested fins as described previously (Sims et al., 2009). To evaluate the relative level of gene expression, whole-mount *in situ* hybridization was completed on four fins in each of three independent trials.

**Table 1.** Primers

		Forward	Reverse
*In situ* hybridization	*cx43*	GCTAGAACTCCCTCAAGATGG	TAATACGACTCACTATAGGGTCCTCTAGCGTTGGGATGTGG
	*ccn2a*	CCAATGACAACCGTGAGTGC	TAATACGACTCACTATAGGGTCCGCCTTCTTAGCTTGGTG
	*evx1*	TAATACGACTCACTATAG	GGATCCATTAACCCTCACTAAAGGGAAGAGCTATGACGTCGCAT
	*axin2*	AGATGACCCACGTCCACCGG	TAATACGACTCACTATAGGGAGAGACACTTGGCCGTTCATCC
q-PCR	*ccn2a*	GGGATCAGCTTTAGCTTAC	AGCATGCGCTCCATTCTGTA
	*ker4*	TCATCGACAAAGTGCGCTTC	TCGATGTTGGAACGTGTGGT
MO verification	MO1	ATTGCTCTGCTGTTCCTGACT	CTGCTAAAGCTGATCCCACAAAG
	MO2	AAAGCCAAACTGTGATGGAAA	AACACATTCCATCCAACAGCA
	Control	TGGTGCCACTGGTGTGTTTG	CTGCTAAAGCTGATCCCACAAAG

### Regenerate length and segment length

Fins were stained with the vital dye calcein before regenerate length and segment length were measured (Du et al., 2001; Sims et al., 2009). Fish swam in 0.2% calcein (pH 7) at room temperature for 1 h and were then returned to fresh water for 10 min. The anesthetized fish were imaged using a Nikon Eclipse 80i Microscope equipped with a SPOT-RTKE digital camera (Diagnostic Instruments) and SPOT software (Diagnostic Instruments). Regenerate length and segment length were measured from the third fin ray from the ventral-most or dorsal-most lobe of the caudal fin (Iovine and Johnson, 2000). Regenerate length was measured from the amputation plane to the distal tip. Segment length was measured as the distance between the first two joints flanking the first complete segment.

### Inhibition of Yap activity

Verteporfin was dissolved in DMSO and was diluted to 20 µM and then injected into the fin using a Narishige IM 300 Microinjector. Fish fins were amputated, and at 3 dpa fish were treated with either Verteporfin or DMSO. Approximately 50 nl of drug was injected per ray into the dorsal rays of the regenerating fin, using the ventral rays as an uninjected internal control. For *in situ* hybridization, fins were harvested at 4 dpa (24 h after drug treatment). For skeletal measurements, fins were treated with calcein at 7 dpa (4 days post treatment).

### Inhibition of β-catenin activity

IWR-1 was dissolved in DMSO and was diluted to 10 µM in 400 ml of system water. ICRT14 was dissolved in DMSO and diluted to 5 µM and then injected into the fin using a Narishige IM 300 Microinjector. Fish fins were amputated and at 3 dpa fish either were treated with IWR-1, ICRT14, or DMSO alone and treated for 24 h. Approximately 50 nl of drug was injected per ray into the dorsal rays of the regenerating tissue, using the ventral rays as an uninjected internal control. At the end of treatment, fins were harvested for *in situ* hybridization.

### qPCR analysis

Trizol reagent (Gibco) was used to extract total RNA from regenerating fins. To prepare cDNA, 1 µg of total RNA was reverse transcribed with SuperScriptIII reverse transcriptase (Invitrogen) using an oligo(dT) primer. Samples from three biological replicates were prepared for all experimental and control conditions. Primer sequences for *ccn2a* and the internal control, *ker4*, can be found in Table 1. Other primer sequences are published (*cx43*, Sims et al., 2009; *axin2* and *evx1*, Bhattacharya et al., 2018). Analyses were performed using the Rotor-Gene 6000 (Corbette Research) and derived C_T_ values using experimental primer pairs and the internal control were averaged. The delta C_T_ (ΔC_T_) values represent expression levels normalized to *ker4* values (Banerji et al., 2017). ΔΔC_T_ values represent the relative level of gene expression. The fold difference was determined using the ΔΔC_T_ method (2^−ΔΔCT^) as described previously (Ton and Iovine, 2013).

### Morpholino-mediated gene knockdown

All morpholinos (MOs) used in the experiments were fluorescein-tagged and purchased from Gene Tools. The MOs were reconstituted in sterile water to 1 mM. The Ccn2a-MO1 (AACAGCCAAGATCCTTACCTGTGCA) is a splice blocking morpholino that binds to the Exon 2 and Intron 2 junction. The Ccn2a-MO2 (AGATTGAGAAATGCTCACCTGCTAA) is a splice blocking morpholino that binds to the Exon 3 and Intron 3 junction. The standard control (SC) MO, (CCTCTTACCTCAGTTACAATTTATA), which does not have any binding sites in the zebrafish genome, was used as a negative control. Microinjection and electroporation procedures were carried out as described previously (Thummel and Iovine, 2017). Briefly, caudal fins were amputated at the 50% level. At 3 days post-amputation (3 dpa), fish were anesthetized and MOs were injected using a Narishige IM 300 Microinjector. Approximately 50 nl of MO was injected per ray into either the dorsal or ventral side of the regenerating fin tissue (the first five or six bony fin rays), keeping the other side uninjected as the internal control. Immediately after injection, both sides of the caudal fin were electroporated using a CUY21 Square Wave electroporator (Protech International). The following parameters were used during electroporation: ten 50 ms pulses of 15 V with a 1 s pause between pulses. After 1 day post-electroporation (1 dpe), which is equivalent to 4 dpa, the injected side of the fins were evaluated by fluorescence using a Nikon Eclipse 80i Microscope (Diagnostic Instruments) to confirm MO uptake. Only fins showing MO uptake were evaluated for regenerate length (7 dpa), segment length (7 dpa), *in situ* hybridization, and RNA levels by qRT-PCR.

### Protein lysate preparation and immunoblotting

Fish fins were amputated at 50% and at 3 dpa fish were treated with 20 µM Verteporfin or DMSO alone for 24 h. At the end of treatment, regenerates were harvested into RIPA lysis buffer and homogenized using a Bio-Gen PRO200 homogenizer at speed setting 3, for 5 s with 10 s cooling. 100µl of 55% TCA was added and tissue was spun at 2000 rpm for 5 min and the protein pellet was subsequently washed with 0.5% TCA. Pellets were dissolved in 100µl of 5× sample buffer and proteins were separated by SDS-PAGE. Following SDS-PAGE, gels were transferred to nitrocellulose. For Yap and Tubulin detection, blots were incubated in 2% BSA in TBST overnight at 4°C, followed by incubation in primary antibody diluted in TBST (Anti-Yap, Cell Signaling Technology; 1A12 mouse mAb used at 1:500; and anti-Tubulin, Sigma-Aldrich, mouse monoclonal T6074 used at 1:1000). Fluorescent secondary antibody anti-mouse Alexa-488 was used at 1:2000 for detection. Band intensities were quantified using ImageJ software. The gel analysis tool in ImageJ, as previously described (Banerji et al., 2017), was used to measure the relative pixel densities of the gel bands. The intensity of each band was determined by calculating the area under the curve. Relative pixel density was calculated as a ratio of Yap normalized to the internal control, tubulin.

### Statistical analyses

Statistical significance was determined using GraphPad Prism software (9.5.1). When comparing two samples a Student's *t-*test (two tailed, unpaired) was performed with Welch's correction. Sample size was determined using a power analysis of 0.8 and a statistical significance of <0.05.
